# Combined analysis of 16S rRNA gene sequencing data reveals core vaginal bacteria across livestock species

**DOI:** 10.3389/fmicb.2025.1524000

**Published:** 2025-02-10

**Authors:** Lucille C. Jonas, Curtis R. Youngs, Stephan Schmitz-Esser

**Affiliations:** ^1^Department of Animal Science, Iowa State University, Ames, IA, United States; ^2^Microbiology Graduate Program, Iowa State University, Ames, IA, United States

**Keywords:** vaginal microbiota, livestock, 16S rRNA, cattle, sheep, pigs

## Abstract

Investigating the livestock vaginal microbiota is of increasing interest due to its relationship with animal reproductive performance. Recent publications have uncovered a high degree of variability of the livestock vaginal microbiota, making it difficult to focus functional research on individual microorganisms. To address this variability, we conducted a combined analysis of publicly available 16S rRNA gene amplicon sequencing datasets to reveal the core vaginal microbiota in cattle, sheep, and pigs. The goal of this combined analysis was to identify bacterial genera that were shared despite a diverse overall sample population. A total of 2,911 vaginal samples (715 cattle, 964 sheep, and 1,232 pigs) from 29 different datasets were used in this combined analysis. Beta diversity analysis revealed structural differences of the vaginal microbiota between different animal species. Compositionally, the most abundant phyla were *Bacillota*, *Pseudomonadota*, and *Bacteroidota.* At the genus level, an unclassified *Pasteurellaceae* genus, *Ureaplasma*, and *Streptococcus* were the most abundant. Across the vaginal microbiota of individual livestock species, compositional differences were observed. The cattle and sheep vaginal microbiota contained a higher abundance of *Ureaplasma* and *Histophilus* whereas the pig vaginal microbiota contained more *Fusobacterium* and *Parvimonas* than that of the other livestock samples. Among the cattle, 120 OTUs and 82 genera were present in 70% of the vaginal samples. At the same threshold, pig samples had 40 core OTUs and 63 core genera, while the sheep samples had 22 core OTUs and 50 core genera. There were 19 overlapping core vaginal genera across the three animal species. The core vaginal OTUs were largely species-specific, although there were eight overlapping OTUs. These included *Streptococcus* (OTU 21), *Clostridium sensu stricto 1* (OTU 18), and *Corynebacterium* (OTU 6), which were also some of the most abundant members of the livestock core vaginal microbiota. A better understanding of the livestock vaginal microbiota is required for future studies aimed at elucidation of the functional significance of individual microbes with respect to livestock reproductive efficiency. The core vaginal genera identified in this analysis will help guide research on mechanisms/pathways through which individual organisms enhance or impede animal reproductive efficiency.

## 1 Introduction

Livestock producers aim to provide high quality animal products at an affordable cost to consumers. In doing so, it is vital for producers to utilize efficient and sustainable management practices. One area of economic loss in livestock production is animal reproductive failure which can result from a variety of causes. Genetics, nutrition, disease, and social status of livestock can influence the fertility of an individual animal (Salak-Johnson, 2017; Bach, 2019). Being able to sustain a pregnancy, withstand parturition, and recover post-partum for subsequent breeding are important for the reproductive efficiency of livestock. Failure to successfully reproduce is a top reason why producers cull animals, whether this is due to infertility, dystocia, or infectious agents causing maladies such as metritis or abortion (Zhao et al., 2015; Workie et al., 2021; Kulkarni et al., 2023). With losses related to reproduction being so prevalent, it is important to investigate different facets of livestock production that may influence reproductive success.

The mammalian reproductive tract harbors diverse commensal microbes that can potentially influence reproductive efficiency. Functionally, it is thought that host vaginal microbiome homeostasis can work to maintain a healthy immune system, while excluding potential reproductive pathogens (Torcia, 2019; Chen et al., 2021). The upset of this environment can cause inflammation of the uterus or vagina, leading to reproductive deficits. Demonstrating the therapeutic potential of this microbial environment, application of intravaginal probiotics significantly decreased the miscarriage rate of women undergoing frozen embryo transfer (Thanaboonyawat et al., 2023). Another recent study demonstrated that the alteration of the sheep vaginal microbiota leads to increased rate of conception with artificial insemination (Toquet et al., 2025).

The livestock reproductive tract microbiota is less understood than that of humans but is of immense interest due to its newfound relationship with reproductive performance (Deng et al., 2019; Sanglard et al., 2020; Koester et al., 2021). Previous investigations have characterized modulations of the vaginal microbiota through different estrous cycle and gestational timepoints (Quereda et al., 2020; Zang et al., 2023; Liu et al., 2025). One study from our laboratory (Cassas et al., 2024) demonstrated clear shifts in the ewe vaginal microbiota throughout gestation, potentially in response to hormonal changes in the host during pregnancy.

More investigations are needed to ascertain the role of the vaginal microbiota in livestock reproduction. Some studies have used culture-dependent methods to characterize the livestock reproductive tract microbiota (Manes et al., 2018; Martinez-Ros et al., 2018; Diop et al., 2019; Webb et al., 2023). Depending on growth conditions and media used, however, culture-based studies often are unable to isolate certain groups of organisms, inadequately covering the complete spectrum of microbial diversity within the reproductive tract. Alternatively, DNA sequencing (specifically, the use of 16S rRNA gene amplicon sequencing) can be employed to examine the composition of these complex microbial communities.

A number of studies investigating the vaginal microbiota present in various livestock species have utilized 16S rRNA sequencing (Kiefer et al., 2021a; Koester et al., 2021; Winders et al., 2023; Zang et al., 2023). Sequencing-based studies have uncovered a high degree of variation in the vaginal microbiota, making it difficult to identify key organisms that may be important for animal reproductive efficiency (Brulin et al., 2024).

The goal of this study was to determine if there are shared vaginal bacteria in livestock species identified through conduct of a combined analysis of previously published 16S rRNA sequencing data. In doing so, this study aims to detect organisms of potential interest for further research to elucidate the physiological mechanisms through which the vaginal microbiota influences livestock reproductive performance.

## 2 Materials and methods

### 2.1 Availability of data and materials

The datasets analyzed in the current study are available in the NCBI Sequence Read Archive (SRA), https://www.ncbi.nlm.nih.gov/sra. Accession numbers to the datasets can be found in Supplementary Table S1.

### 2.2 Sequencing data collection and initial quality control

A literature review was conducted for studies investigating the vaginal microbiota of livestock species using 16S rRNA amplicon sequencing. Publications were identified through searching the NCBI SRA, PubMed, and Google Scholar. Key search terms included “livestock vaginal microbiota,” “livestock vaginal microbiome,” and “16S sequencing.”

A total of 50 studies was identified for potential inclusion in this study. To be included in the analysis, sequencing of the variable region 4 (V4) of the 16S rRNA gene must have been done using an Illumina platform and yielding at least 10,000 reads after initial quality control. The inclusion of sequences of the same variable region allows for superior comparison of the vaginal microbiota from different studies as it facilitates *de novo* operational taxonomic unit (OTU) clustering. Only vaginal samples from weaned animals were included in this analysis because of the assumption that the vaginal microbiota experiences a level of instability during early life, similar to that seen in the gut microbiota (Du et al., 2023). Studies involving non-vaginal samples or process controls had to include meaningful metadata in their SRA submission to distinguish between sample types. Sequences were downloaded using the NCBI sra-toolkit.

Of the 50 studies considered for this analysis, 14 were excluded because they did not publish their raw sequencing data on the NCBI SRA. Another five studies were excluded due to concerns with their SRA submissions (missing/incorrect metadata or pre-assembled contigs). Three studies were excluded because sequencing was not performed using an Illumina platform. One study was excluded because it sequenced the V1-3 region of the 16S rRNA gene, making it incompatible with *de novo* OTU clustering (Clemmons et al., 2017). Yet another study was excluded because it sampled the vaginal microbiota of newborn calves (Luecke et al., 2023). Finally, one additional study was excluded because samples had less than 10,000 reads after quality control (Tasara et al., 2023).

In addition to the remaining 25 publications described above, unpublished livestock vaginal sequencing data from four studies conducted through Iowa State University (ISU) were also used for this study. Of the 29 studies included in the final analysis, 13 sampled the pig vaginal microbiota, 10 sampled cattle, and 6 sampled sheep. Samples were collected from animals located in 8 different countries, and studies investigated a variety of experimental variables such as breed, parity, and mating strategy [natural service or artificial insemination (AI)]. Most vaginal microbiota samples were collected by using a vaginal swab, although one study with pigs (Alves et al., 2022) used a vaginal lavage to collect samples. Some studies (Poor et al., 2022; Diaz-Lundahl et al., 2023; Mariadassou et al., 2023) involved more invasive instruments during sampling, such as a speculum or MetriCheck instrument. The different sampling procedures are of note because they could inadvertently introduce biases into the vaginal communities recovered. An overview of the studies from which samples were used in this analysis can be found in Supplementary Table S1.

### 2.3 Sequencing data analysis

The mothur v1.48.0 software was used for sequence quality control and processing (Schloss et al., 2009). Paired-end reads were assembled with the “make. Contings” command and were screened for quality by total sequence length, ambiguities, and homopolymer length. Subsampling was conducted to avoid bias toward studies with higher sequencing depth. Samples were subsampled to 10,000 sequences using the “sub. Sample” command with the “persample” option (Schloss et al., 2009). Sequences were aligned to a trimmed SILVA SSU database (V138) (Quast et al., 2013) generated with “pcr.seqs” in mothur so that all sequences were solely that of the V4 region. After the alignment, chimeric sequences were removed by using the “chimera.vsearch” command with the SILVA-gold reference database provided by the mothur website. *De novo* OTU clustering was conducted at 97% similarity using the “cluster.split” command in mothur, and sequences were classified with the SILVA SSU database. Representative sequences of selected OTUs were further identified using the NCBI Basic Local Alignment Search Tool (BLAST).

Further analysis was conducted in R v4.3.1 (R Development Core Team, 2010) in R studio using phyloseq (McMurdie and Holmes, 2013) and microbiome packages (Lahti and Shetty, 2017). Continued quality control was conducted by removing samples with low sequencing depth (<5,000 sequences), along with OTUs that had fewer than 10 reads. To assess differences in vaginal community structure, samples were normalized and Bray-Curtis distances were calculated. Beta diversity was then visualized using principle coordinate analysis graphs. Additionally, permutational multivariate analysis of variance (PERMANOVA) and dispersion (PERMDISP2) of the Bray-Curtis dissimilarities was assessed with the adonis2 and betadisper commands from the VEGAN package (Oksanen et al., 2024). Vaginal sample microbiota composition was calculated using sequence abundances of phyla, genera, and OTUs. All figures were generated using ggplot or eulerr (Wickham, 2016; Larsson et al., 2024). Core vaginal microbes were calculated with genera or OTU prevalence across a population of samples. In this study, a genus was considered part of a core if it was found in at least 70% of the samples in a group. Core vaginal genera and OTUs were calculated across all samples included in this analysis, along with separate cores among samples within the same livestock species.

## 3 Results

### 3.1 Overview of dataset

A total of 3,235 vaginal microbiota samples from 29 studies (including the four unpublished ISU datasets) with raw 16S rRNA amplicon sequencing data were included in the initial analysis. Prior to alignment to the trimmed V4 reference, 281 samples were removed due to insufficient sequencing depth (<10,000 reads). After alignment to the 16S rRNA V4 region, chimera removal and further quality control, samples with less than 5,000 reads (*n* = 43) were removed. The final dataset comprised of 2,911 samples and contained a combined total of 27,244,861 sequences, having an average sequencing depth of 9,359 reads per sample with a standard deviation of 601 sequences. Of the 2,911 samples, 715 were from cattle (10 studies), 964 were from sheep (6 studies), and 1,232 were from pigs (13 studies).

**Figure 1:**
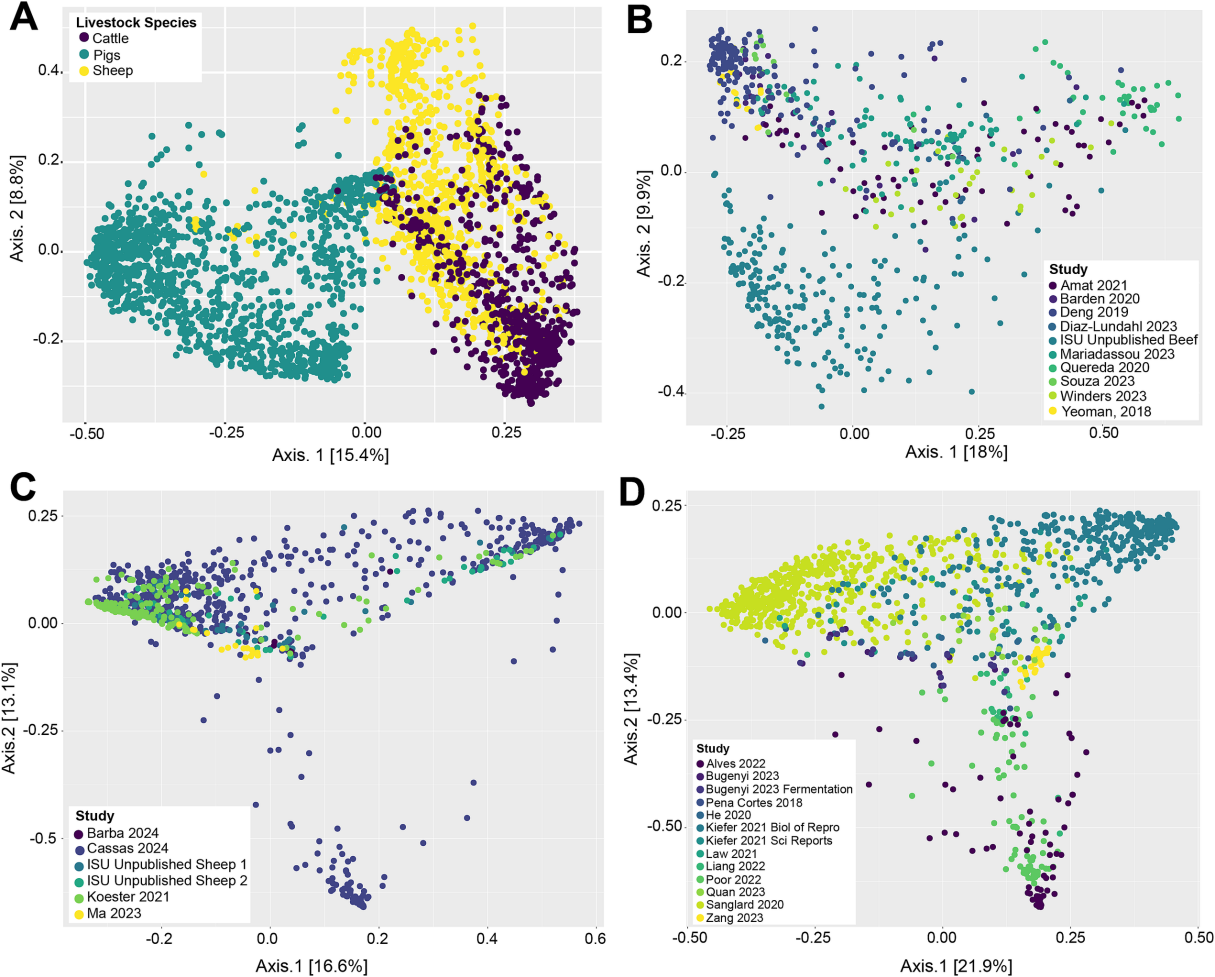
Principal coordinate analysis (PCoA) based on Bray–Curtis dissimilarity measures of the vaginal microbial community structure of all livestock samples included in this analysis **(A)**, cattle samples **(B)**, sheep samples **(C)**, and pig samples **(D)**.

Beta-diversity of livestock vaginal microbiota was analyzed with Bray-Curtis distances and visualized as principle coordinate analysis (PCoA) plots (Figure 1). Figure 1A illustrates the differences in vaginal microbial community structure across the different livestock species (cattle, sheep, and pigs) in this study. Visually, there appeared to be separate clustering between the pig vaginal microbiota and that of samples taken from ruminants (sheep and cattle), along with a degree of separation between cattle and sheep samples (Figure 1A). This suggests that the vaginal microbiota is structurally different between the three animal species examined. A significant PERMANOVA (*p* = 0.001) of the Bray-Curtis distances demonstrated that animal species had a potential effect on the microbial community structure. However, for the same variable, the PERMDISP2 was also significant (*p* = 0.001), indicating unequal dispersions of the Bray-Curtis distances for the different animal species. Figure 1B depicts the vaginal microbial community structure across samples taken from cattle. The vaginal microbial community structure of cattle appears to be somewhat homogenous, as samples from different studies were mostly intermixed. There was separation of some samples in the ISU unpublished beef dataset from the remainder of the cattle samples, however, indicating that they have different vaginal community structure. Visually, the vaginal microbial community structures of sheep (Figure 1C) and pig samples (Figure 1D) were mostly intermixed, lacking any distinctive clustering dependent on the study. The PERMANOVA based on study origin was significant (*p* = 0.001); however again, the PERMDISP2 was also significant (*p* = 0.001).

**Figure 2:**
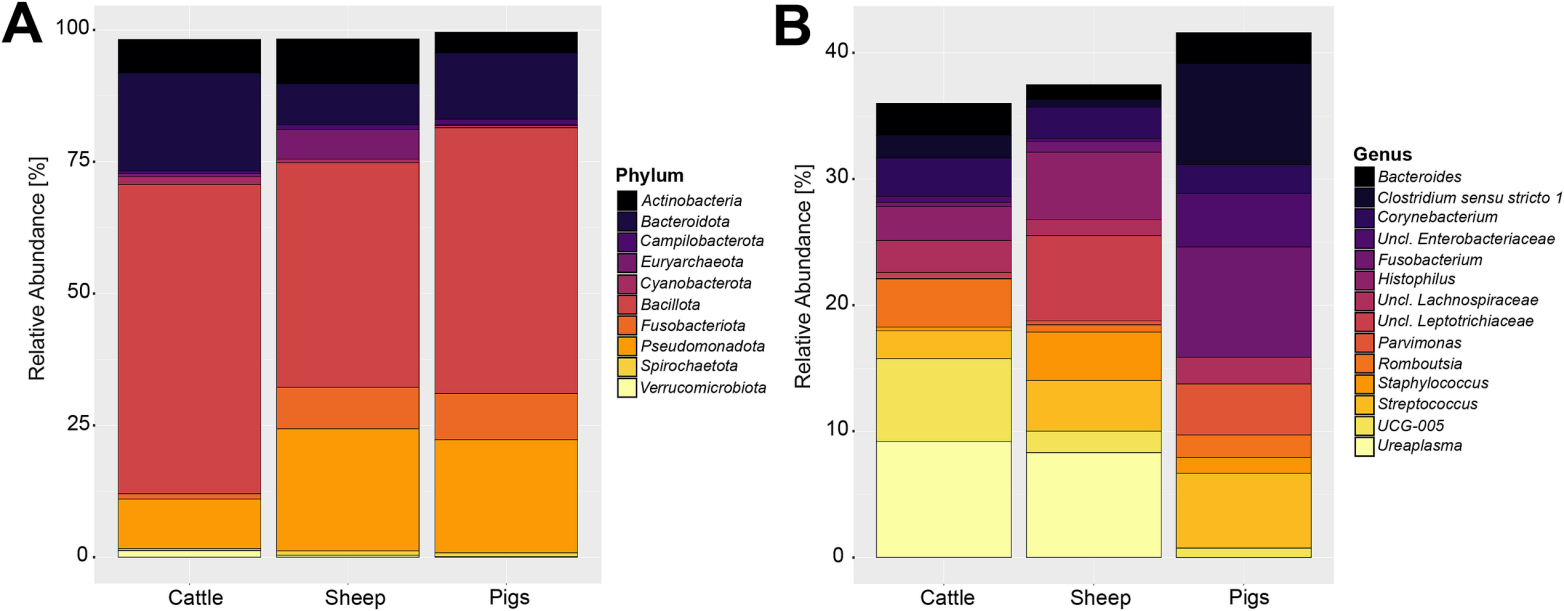
Relative abundance of the 10 most abundant bacterial and archaeal phyla **(A)** and 15 most abundant genera **(B)** in the livestock vaginal microbiota. “Unclassified” was abbreviated as “Uncl.,” and refers to unclassified genera within the respective families.

Compositionally, the 27,244,861 compiled sequences were assigned to 59 phyla, 2,174 genera, and 23,773 OTUs. Supplementary Table S2 contains a detailed overview of the 10 most abundant phyla across the three livestock species included in this analysis. The most abundant phylum was *Bacillota* (previously: *Firmicutes*), proportionally making up 49.79% of the sequences. The second and third most abundant phyla were *Pseudomonadota* (previously: *Proteobacteria*) and *Bacteroidota* (previously: *Bacteroidetes*) making up 19.11 and 12.34% of the reads, respectively. These three most abundant phyla accounted for 81.24% of all sequences. The phylum-level composition varied slightly among the three livestock species (Figure 2A). The sheep and pig vaginal microbiota contained higher relative abundances of *Fusobacteriota* and *Pseudomonadota*, compared with cattle. Other notable differences were that the sheep vaginal samples contained more *Cyanobacteriota* than those of cattle and pigs, whereas the cattle samples had higher relative abundances of *Bacteroidota* than sheep or pigs.

The most abundant genus throughout all samples was an unclassified *Pasteurellaceae* genus, making up 8.30% of sequences. Using NCBI BLAST, the most abundant OTU belonging to the unclassified *Pasteurellaceae* genus (OTU 2) was classified as *Actinobacillus porcinus* (98.8% identity). *Ureaplasma, Streptococcus*, and *Fusobacterium* were the second, third, and fourth most abundant genera across all samples, making up 5.05, 4.36, and 4.17% of all sequences, respectively. The breakdown of the 15 most abundant genera across all vaginal samples by livestock species can be seen in Figure 2B. At the genus level of classification, the sheep and cattle vaginal microbiota appeared more similar to each other than that of the pig. There were higher relative abundances of *Ureaplasma, Oscillospiraceae UCG-005*, and *Histophilus* in sheep and cattle compared with pigs. In contrast, the pig vaginal microbiota contained higher relative abundances of *Fusobacterium* and *Bacteroides* compared with the ruminant livestock species. Supplementary Table S3 contains the relative abundances of the 50 most abundant genera for each livestock species included in this analysis.

While examining the most abundant genera across the entire dataset is valuable for comparison of vaginal microbiota among different livestock species, comparison of samples within a species across studies is also important. Supplementary Figures S1–S3 depict the 15 most abundant genera within the cattle, sheep, and pig samples, respectively. In these figures, variation of the genus composition in the vaginal microbiota within individual livestock species can be seen.

### 3.2 Core vaginal microbiota

To be considered as a core bacterial genus in this analysis, a genus or OTU needed to be present in at least 70% of the samples within a given population. Among the 2,911 samples analyzed, there were 44 genera and 14 OTUs that were present in at least 70% of the samples. Cattle samples contained 120 core OTUs that were at least 70% prevalent (Supplementary Table S4), pig samples had 40 core OTUs (Supplementary Table S5), and sheep had only 22 core OTUs (Supplementary Table S6). The repeated nature of the genus classifications of the core OTUs is an indicator of diversity within the vaginal microbiota of livestock. For instance, the cattle share 13 core OTUs that were within the *Oscillospiraceae* UCG-010 genus. The most abundant genus within the pig core OTUs was *Clostridium sensu stricto 1*, and *Streptococcus* within the sheep core OTUs. The core OTUs were largely animal-specific; however, cattle, sheep, and pigs shared eight overlapping vaginal OTUs (Supplementary Table S7), all being relatively abundant across the entire dataset. The most abundant vaginal core OTU was *Corynebacterium* (OTU 6) (1.90%) relative abundance, followed by *Romboutsia* (OTU 8) (1.88%), and *Turicibater* (OTU 12) (1.36%).

**Figure 3:**
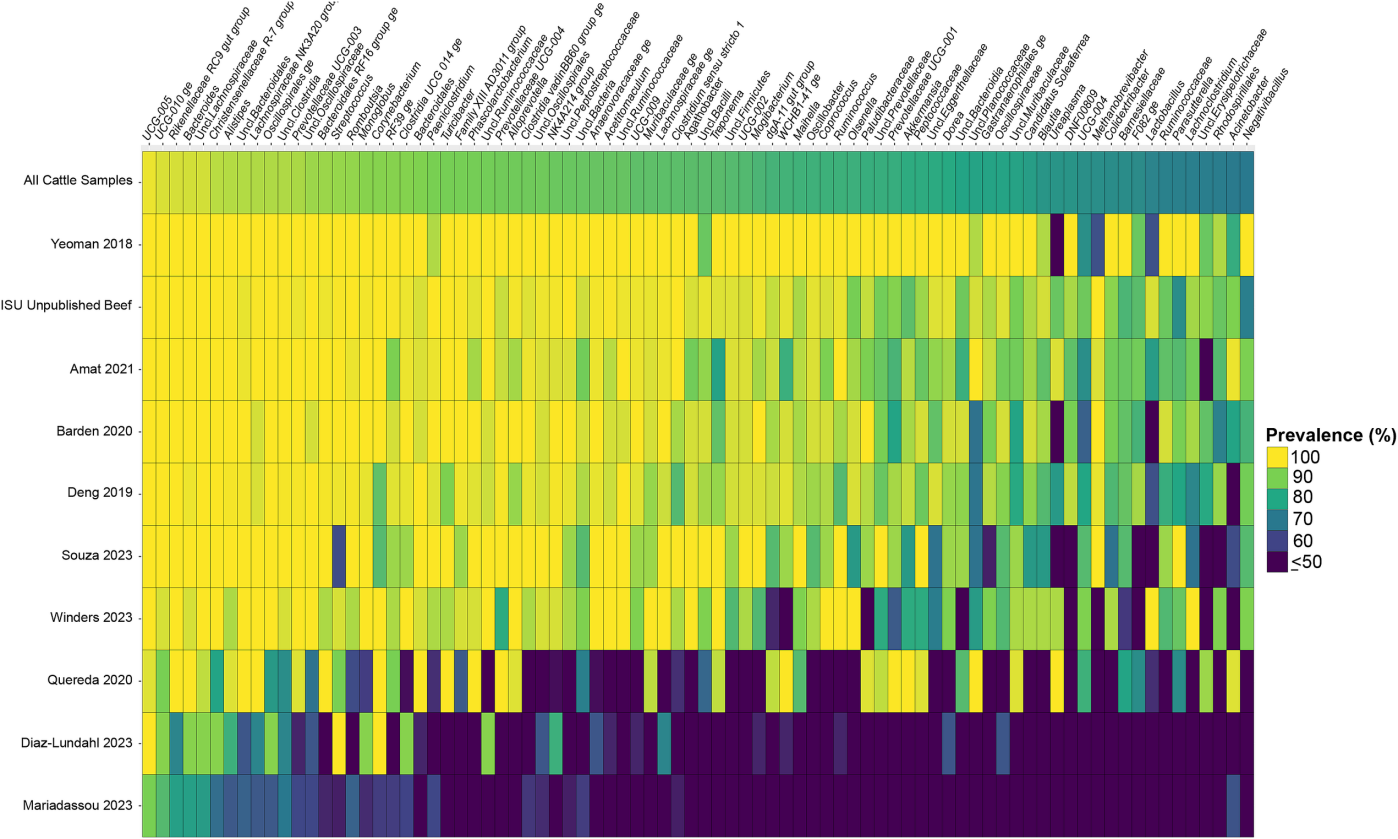
Heatmap of the prevalence of the 82 core vaginal genera in cattle by study from which the samples were derived. Studies are ordered by average prevalence of the core genera. Prevalence less than 50% was truncated to provide greater resolution for the higher values. “Unclassified” was abbreviated as “Uncl.,” and refers to unclassified genera within the respective families.

**Figure 4:**
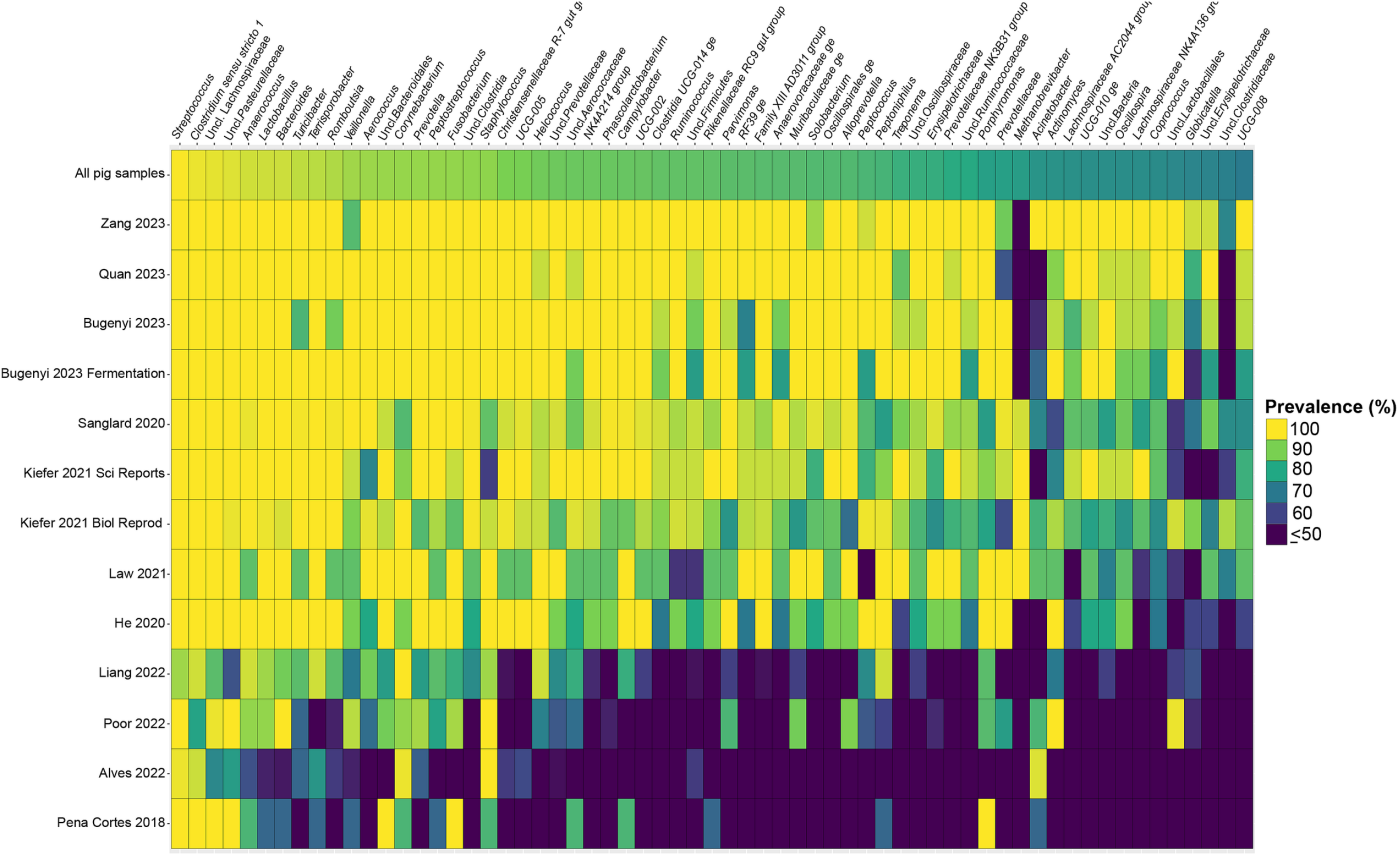
Heatmap of the prevalence of the 63 core vaginal microbial genera in pigs by study from which the samples were derived. Studies are ordered by average prevalence of the core genera. Prevalence less than 50% was truncated to provide greater resolution for the higher values. “Unclassified” was abbreviated as “Uncl.,” and refers to unclassified genera within the respective families.

**Figure 5:**
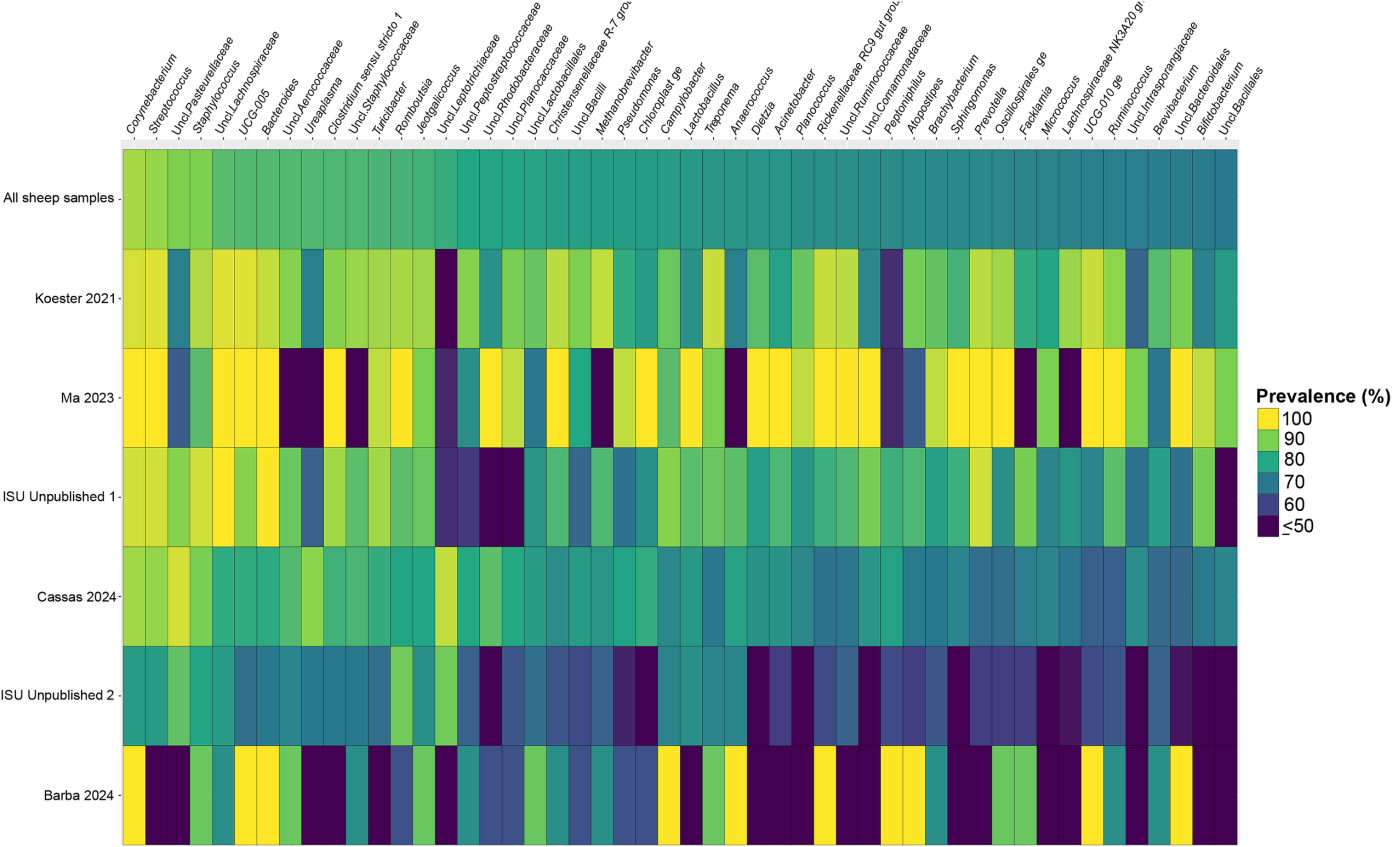
Heatmap of the prevalence of the 50 core vaginal microbial genera in sheep by study from which the samples were derived. Studies are ordered by average prevalence to the core genera. Prevalence less than 50% was truncated to provide greater resolution for the higher values. “Unclassified” has been abbreviated to “Uncl.,” and refers to unclassified genera within the respective families.

**Figure 6:**
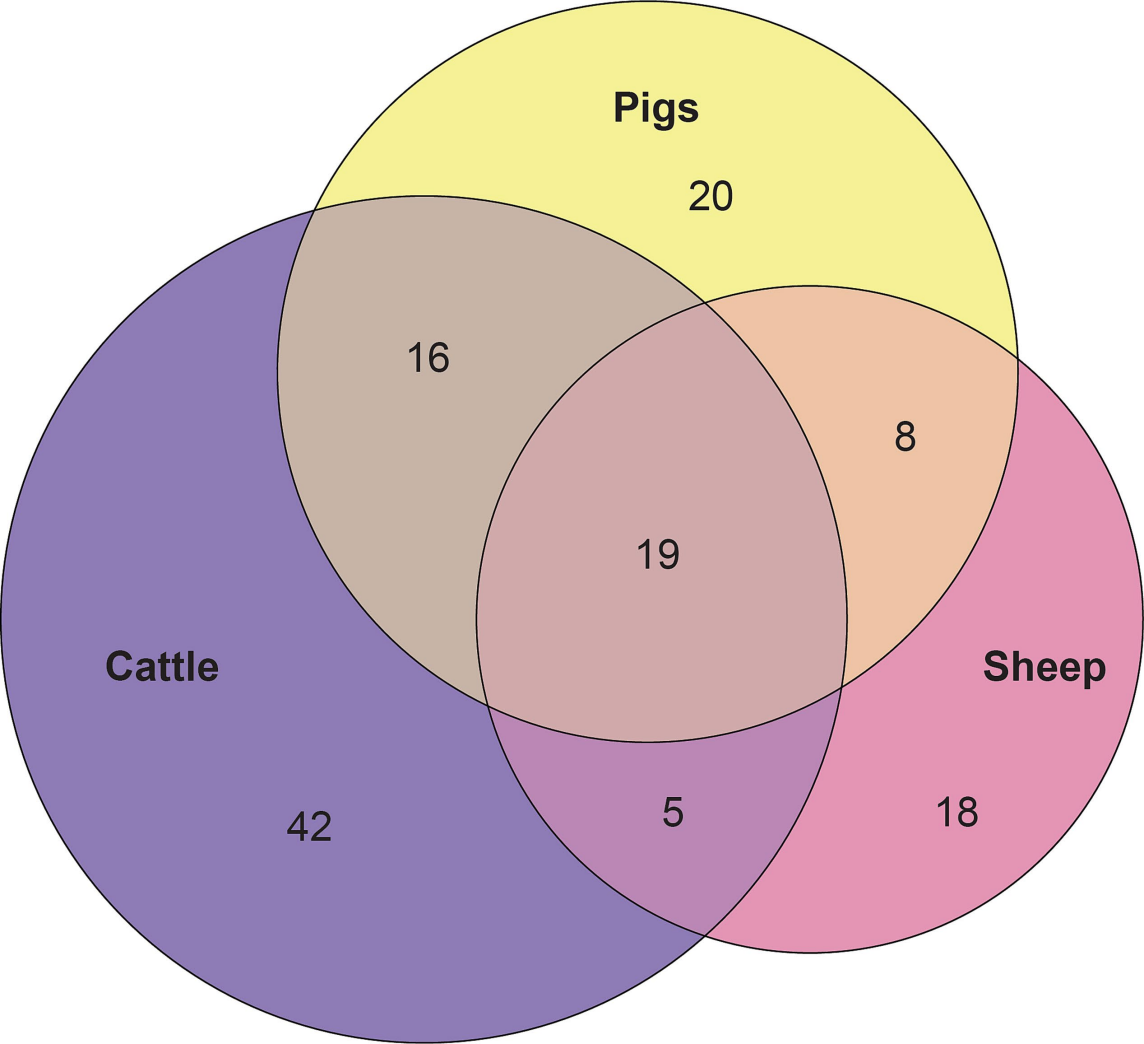
Euler diagram depicting the 19 overlapping core vaginal genera of cattle (82 genera), sheep (50 genera), and pigs (63 genera).

Considering the genus level of classification, cattle vaginal samples had an individual core of 82 genera (Figure 3), pig samples had a core of 63 genera (Figure 4), and sheep samples had a core of 50 genera (Figure 5). Within the cattle vaginal core microbiota 60 genera (73%) were at least 80% prevalent, and in the pig vaginal core microbiota 47 genera (74%) fell within the same threshold. However, within the sheep vaginal core microbiota only 15 genera (30%) were 80% prevalent or greater. The overlapping genera of the three separate livestock cores shown in Figure 6 illustrate the overall livestock core vaginal microbiota. Between the ruminant species in this study, sheep and cattle uniquely shared five core vaginal genera. Cattle and pigs uniquely shared 16 core genera, and sheep and pigs shared eight unique core vaginal genera. Across the three species, there were 19 genera that were at least 70% prevalent within their respective species’ pool of samples. The shared 19 core genera are depicted in Table 1, along with the corresponding relative abundances in the different species’ samples.

**Table 1:** Relative abundance (%) of the 19 core vaginal microbiota genera in livestock.

Classification	All samples (*n* = 2,911)	Cattle samples (*n* = 715)	Sheep samples (*n* = 964)	Pig samples (*n* = 1,232)
*Streptococcus*	4.36	2.19	4.01	5.85
*Clostridium_sensu_stricto_1*	4.07	1.87	0.60	8.02
*Corynebacterium*	2.53	3.05	2.46	2.30
*Oscillospiraceae UCG-005*	2.44	6.48	1.72	0.74
*Bacteroides*	2.03	2.49	1.16	2.47
Unclassified *Lachnospiraceae*	1.93	2.50	1.26	2.14
*Romboutsia*	1.91	3.96	0.58	1.80
*Turicibacter*	1.38	0.79	0.74	2.21
*Oscillospirales UCG-010_ge*	1.36	4.84	0.45	0.14
*Rikenellaceae_RC9_gut_group*	1.17	3.36	0.60	0.40
*Christensenellaceae_R-7_group*	0.99	2.19	0.87	0.41
*Lactobacillus*	0.75	0.47	0.42	1.17
*Methanobrevibacter*	0.72	1.36	0.63	0.44
*Oscillospirales_ge*	0.71	2.00	0.38	0.25
*Treponema*	0.61	0.34	0.75	0.66
Unclassified *Bacteroidales*	0.58	1.02	0.28	0.58
*Acinetobacter*	0.55	0.41	0.52	0.64
Unclassified *Ruminococcus*	0.40	0.76	0.46	0.16
*Ruminococcus*	0.25	0.17	0.26	0.28

## 4 Discussion

The aim of this comprehensive combined analysis was to reveal microorganisms of potential interest in the livestock vaginal microbiota through systematically comparing 16S rRNA sequencing data from a diverse array of studies. Previous reviews of the livestock reproductive tract microbiota (mainly regarding cattle) have been published (Adnane and Chapwanya, 2022; Luecke et al., 2022; Poole et al., 2023); however, this is the first combined sequence analysis of published reproductive tract microbiota amplicon sequencing data. Our study is similar in scope to previous meta-analyses using 16S rRNA amplicon sequencing data to identify patterns and core microbiota from diverse arrays of livestock gastrointestinal tract samples (Holman et al., 2017; Holman and Gzyl, 2019; Anderson et al., 2021; Pacífico et al., 2021). Those previous 16S rRNA gene meta-analyses have helped focus ongoing research of the livestock gut microbiota because core bacteria that remain prevalent, despite a diverse sample population, implies potential biological significance of said bacteria. The same theory applies to the livestock reproductive tract because bacteria that are consistently found in the vagina across species may have possible implications on reproductive performance.

### 4.1 Limitations

An important note about the microbial classifications discussed throughout this study is that the majority of them belong to unclassified organisms, some are even referred to by their family-level classification as opposed to their genus. This highlights a general limitation of 16S rRNA amplicon sequencing because the microbial classifications derived from this technology rely on a short sequence of DNA (V4 ~ 250 bp), and, therefore, taxonomic resolution is limited (Weinroth et al., 2022). Furthermore, the livestock reproductive tract microbiota is comparatively understudied to other anatomic communities, making it less likely that the classification databases (SILVA SSU and NCBI) have accurate references for the organisms within this environment. Nevertheless, the continued investigation of the livestock vaginal microbiota can help direct future research to bridge gaps in the current body of knowledge. Another limitation with using DNA sequencing as a means to examine the vaginal microbiota of livestock is that the reproductive tract is low in microbial biomass, making vaginal samples more at risk for contamination. While it is known that there are overlapping bacteria that are found in multiple anatomic locations (Bugenyi et al., 2023; Mariadassou et al., 2023), when considering the location of the vaginal canal in livestock it is possible that DNA from fecal microbes might contaminate some of the vaginal samples. By identifying conserved organisms across a variety of studies, it is assumed that core vaginal microbes are less likely to be purely fecal contaminants.

This investigation considered the vaginal microbiota of three livestock species: cattle, sheep, and pigs. While the main goal of this study was comparing the similarities between the animal species, the variable nature of the vaginal microbiota should be addressed. Inconsistencies of the vaginal microbiota across individual animals was a challenge researchers faced when attempting to characterize this system. One study (Quereda et al., 2020) aimed to evaluate the cow vaginal microbiota between the follicular and luteal phases of the estrous cycle. Even though heifers were used in that study to avoid potential biases from breeding, parturition and post-calving infections, there were remarkable differences in the vaginal microbiota across individuals at the comparable timepoints. The same phenomenon can be seen in one study (Chen et al., 2020) characterizing the vaginal microbiota of cows at the time of AI, where the comparison between the resultant pregnant and open cows was difficult due to the inconsistent vaginal microbiota across animals. Another recent study characterizing the vaginal microbiota of 19 French dairy cattle herds also revealed a high variability of the vaginal microbiota (Brulin et al., 2024). The variation of this system is further illustrated in Supplementary Figures S1–S3, which examine the microbial community abundances of the cattle, sheep, and pig vaginal samples from different investigations.

To accomplish the goal of this analysis, a diverse population of samples was required to uncover the prevailing core microorganisms. In doing so, the diversity of this compiled dataset also means it is impossible to draw conclusions about variation in vaginal community structure and composition with the conditions used in the individual investigations (nor does this align with the aim of this analysis). By embracing the diverse nature of the sample population (including samples from animals at different parities, breeds, diets, geographic location, and mating strategies), the organisms that remain highly abundant and prevalent are interesting because of their possible connection to reproductive efficiency. The core microbes discovered here will aid future researchers to navigate the established variation in pursuit of elucidating the specific role of the vaginal microbiota on livestock reproduction.

Another note on variation is that when examining the separate animal vaginal cores, it is evident that core genera are not equally prevalent among studies. Of most note are the investigations with little prevalence of the core genera. Specifically, one study (Diaz-Lundahl et al., 2023) only had 18 of the cattle core genera present in more than 70% of its samples, whereas another study (Mariadassou et al., 2023) only had six genera in line with the cattle vaginal core. This is in contrast to another study included in the cattle dataset (Quereda et al., 2020) in which samples also appeared to fit in less with the cattle vaginal core, but still had 42 of the genera being at least 70% prevalent. The same phenomenon was observed with the pigs where the samples from four studies (Pena Cortes et al., 2018; Alves et al., 2022; Liang et al., 2022; Poor et al., 2022) had less prevalence of the pig core vaginal microbiota compared to the rest of the pig studies.

As mentioned previously, the majority of the vaginal samples used in this analysis were taken via a vaginal swab; however, a few studies utilized different sampling techniques and processing. Two studies (Poor et al., 2022; Mariadassou et al., 2023) involved samples taken with vaginal swabs but employed a speculum to facilitate the sampling. Samples were taken via vaginal lavage in one study (Alves et al., 2022), while yet another study (Diaz-Lundahl et al., 2023) used a MetriCheck device during the sampling period. Given that the samples from these studies overall had lower prevalence of their respective core vaginal microbes, one could speculate that the more invasive sampling procedures altered the vaginal communities recovered. On this note, pig vaginal swabs in one study (Pena Cortes et al., 2018) were subsequently placed into 80% ethanol, which again potentially altered the vaginal communities identified via DNA sequencing. There could be a number of factors contributing to vaginal community makeup, but future studies should take the findings of this analysis into account when determining vaginal sampling procedures.

### 4.2 The core vaginal microbiota of livestock

This combined analysis uncovered overlapping core vaginal microbiota within and across different livestock species. At the OTU-level, there was little overlap between the cores of cattle, sheep, and pigs. However, on an individual basis, the comparison of the species’ core OTUs and genera provides insights into the diversity of the vaginal microbiota. Multiple core OTUs sharing the same genus classification indicates that multiple species or strains of said genus may be conserved in the vaginal microbiota. The cattle samples had the largest core of OTUs, along with the most taxonomic redundancy. Specifically, many cattle core OTUs belonged to unclassified *Clostridia* genera (within the *Oscillospiraceae, Christensenellaceae, Lachnospiraceae* families). The same can be said, albeit to a lesser extent, for the pig core vaginal OTUs. The reverse, genera present in the genus core but not in the OTU core are also interesting. For instance, 37 of the 82 cattle core genera did not have specific core OTUs, implying that sequences of said genera were more diverse from sample to sample. The same goes for 35 genera of the 63 core pig genera, and 30 of the 50 sheep core genera. The sheep overall had the smallest vaginal cores (OTU and genus), demonstrating a high degree of variation across individuals. The diversity of the vaginal microbiota in sheep could be explained by differences in animal management practices. Sheep samples included in this study were more likely to be taken from animals who were reared in grazing systems and bred via natural service, both of which are less controlled than commercial operations of cattle or pigs.

The overall livestock core vaginal microbiota was comprised of 19 genera that remained highly prevalent (>70% prevalence) across the 2,911 samples from three different animal species. Eight of these genera had corresponding overlapping core OTUs, which comprised the majority of their prevalence and abundance. The core vaginal genera that were consistently abundant across cattle, sheep, and pig samples were *Streptococcus*, *Corynebacterium*, and *Bacteroides*; all three are commonly regarded as pathogenic in and outside of the reproductive tract. Cultivation-based studies found that the presence of *Streptococcus* spp. is correlated with uterine health status and inflammation in pigs and cattle (Wang et al., 2020; Ballas et al., 2023), and *Streptococcus equi* is an especially well-characterized pathogen causing bacterial endometritis in mares (Li et al., 2021). *Streptococcus* was also found more abundant in ewes who failed to become pregnant (Koester et al., 2021) and in sows with higher risk of pelvic organ prolapse (Kiefer et al., 2021b). Conversely, several other studies found high relative abundances of *Streptococcus* in healthy sheep, cattle, and pig vaginal samples (Liang et al., 2022; Souza et al., 2023; Cassas et al., 2024).

*Corynebacterium* is similar to *Streptococcus* in that it is also associated with disease in animals. For example, *Corynebacterium pseudotuberculosis* can cause caseous lymphadenitis in sheep and goats, leading to lesions in the reproductive tract (Othman et al., 2016). It is likely that the core vaginal *Corynebacterium* does not harbor the same pathogenicity as *C. pseudotuberculosis* because the representative sequence for the most abundant *Corynebacterium* OTU (OTU6) was classified as *Corynebacterium casei* (99.6% identity) when using NCBI BLAST. The effects of *Corynebacterium* on reproductive performance are unclear. One study in dairy cattle found that *Corynebacterium* was more abundant in the uterus of healthy cows than in those with mild endometritis (Ballas et al., 2021). In contrast, another study (Poor et al., 2022) found that *Corynebacterium* was positively correlated to *Prevotella* in sows with purulent vaginal discharge. While there were overlapping core OTUs classified as *Corynebacterium* (OTU 6) and *Streptococcus* (OTU 21), there were no overlapping core *Bacteroides* OTUs, indicating there was more diversity within *Bacteroides* sequences. *Bacteroides* is also thought to be a driver of endometritis in cattle and pigs (Wang et al., 2017; Adnane and Chapwanya, 2022).

Unclassified *Pasteurellaceae* was the most abundant core vaginal genus in pigs and second most abundant core vaginal genus in sheep. The representative sequence for the most prevalent Unclassified *Pasteurellaceae* OTU in pigs was *Actinobacillus porcinus* while the same for sheep was *Actinobacillus seminis* (both 98.8% identity) when using NCBI BLAST. While *Pasteurellaceae* was not conserved enough to be in the cattle core vaginal microbiota, *Histophilus* was present in samples from certain studies (Quereda et al., 2020; Mariadassou et al., 2023; Souza et al., 2023; Winders et al., 2023). Members of the *Pasteurellaceae* family are commonly found in the healthy ewe reproductive tract (Koester et al., 2021; Greenwood et al., 2022; Barba et al., 2024; Cassas et al., 2024), despite being generally regarded as pathogenic. This view is in part due to some strains’ ability to cause respiratory illness in cattle and sheep (Brogden et al., 1998; Harper et al., 2006; Horiguchi, 2012). However, the combined high prevalence and abundance of these organisms in the livestock vaginal microbiota calls for further research into the contextual pathogenesis of *Pasteurellaceae.* Like many Gram-negative pathogens, the lipopolysaccharide (LPS) from *Pasteurellaceae* contributes to its virulence, among other adhesins, secretion systems, and capsules (Bojesen et al., 2022; Bossé et al., 2022; Caswell and Czuprynski, 2022; Harper et al., 2022). Given the prevalence of *Pasteurellaceae* in the vaginal communities of healthy livestock, more investigation is warranted for the commensal isolates, particularly regarding the presence or absence of certain virulence factors.

Another interesting member of the livestock vaginal microbiota, and the most abundant genus within the cattle and sheep core, was *Ureaplasma* (OTU1)*. Ureaplasma* was not present in the pig vaginal core, suggesting that it may have a tropism for ruminant reproductive physiology. *Ureaplasma* is often regarded as a pathogen due to its ability to cause inflammation of the reproductive tract (Doig et al., 1980a,b). Moreover, *Ureaplasma* has been shown to change prostaglandin E2 and F2α synthesis of endometrial cells (Kim et al., 1994), which could alter the reproductive status of an animal. The overall high abundance and prevalence of *Ureaplasma* among ruminant vaginal samples can possibly be attributed to the urogenital physiology of mammalian livestock because urea, derived from urine, is used in the metabolism of *Ureaplasma* to produce ATP (Smith et al., 1993). Variations in breed anatomy or sampling location could explain differing levels of *Ureaplasma* in the livestock vaginal microbiota. Linking back to its utilization of urea hydrolysis in ATP synthesis, the production and effect of the ammonia on the vaginal environment is interesting. The human vaginal microbiome, while vastly different to that of livestock, relies on activity of lactic acid bacteria to maintain a low pH which inhibits the colonization of incoming pathogens and overgrowth of commensal inhabitants (Miller et al., 2016). In contrast to humans, ewe and cattle vaginal lavages reveal a close to neutral environment (Swartz et al., 2014), so the role of *Ureaplasma* as a core member of the ruminant livestock vagina on other community members and reproductive efficiency is unclear.

## 5 Conclusion

This study is the first combined sequence analysis to elucidate the core vaginal microbiota of livestock through systematically conglomerating 16S rRNA gene sequencing data from previously published works. By purposely including samples taken from animals with different variables (breeds, parities, geographic location), the strength of the core vaginal microbiota discovered in this analysis is maximized because these genera are broadly prevalent despite diverse conditions, implying potential biological significance. Little is known about the functionality of most of the core vaginal microorganisms highlighted in this analysis. This lack of information can be partially attributed to cultivation difficulties because many of these bacteria are greatly fastidious and survive poorly outside of their host, making DNA sequencing studies some of the first records of their existence in the livestock vaginal microbiota. Future efforts in this field must extend beyond 16S rRNA gene amplicon sequencing and into techniques that allow for the more complete investigation of the core vaginal microorganisms reported in this analysis.

## Data Availability

The original contributions presented in the study are included in the article/Supplementary material, further inquiries can be directed to the corresponding author.
