# Supplementary material for: Combined analysis of 16S rRNA gene sequencing data reveals core vaginal bacteria across livestock species
Source: Front Microbiol. 2025 Feb 10;16:1524000. doi: 10.3389/fmicb.2025.1524000 (PMC11849051; doi:10.3389/fmicb.2025.1524000)
Supplement: Supplementary file 1 [file Image_1.PDF]

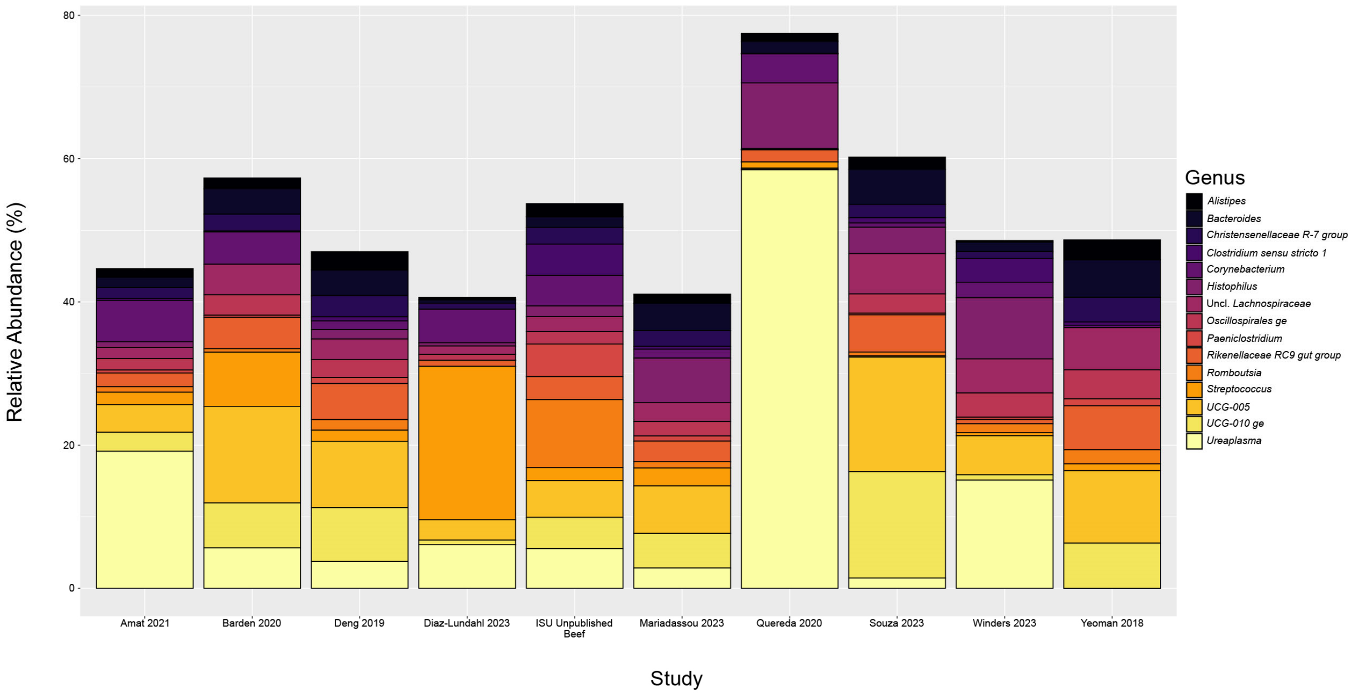


**Additional file 4: S1 Figure:** Relative abundance of the 15 most abundant genera in the cattle vaginal microbiota.
