# Supplementary material for: Combined analysis of 16S rRNA gene sequencing data reveals core vaginal bacteria across livestock species
Source: Front Microbiol. 2025 Feb 10;16:1524000. doi: 10.3389/fmicb.2025.1524000 (PMC11849051; doi:10.3389/fmicb.2025.1524000)
Supplement: Supplementary file 2 [file Image_2.PDF]

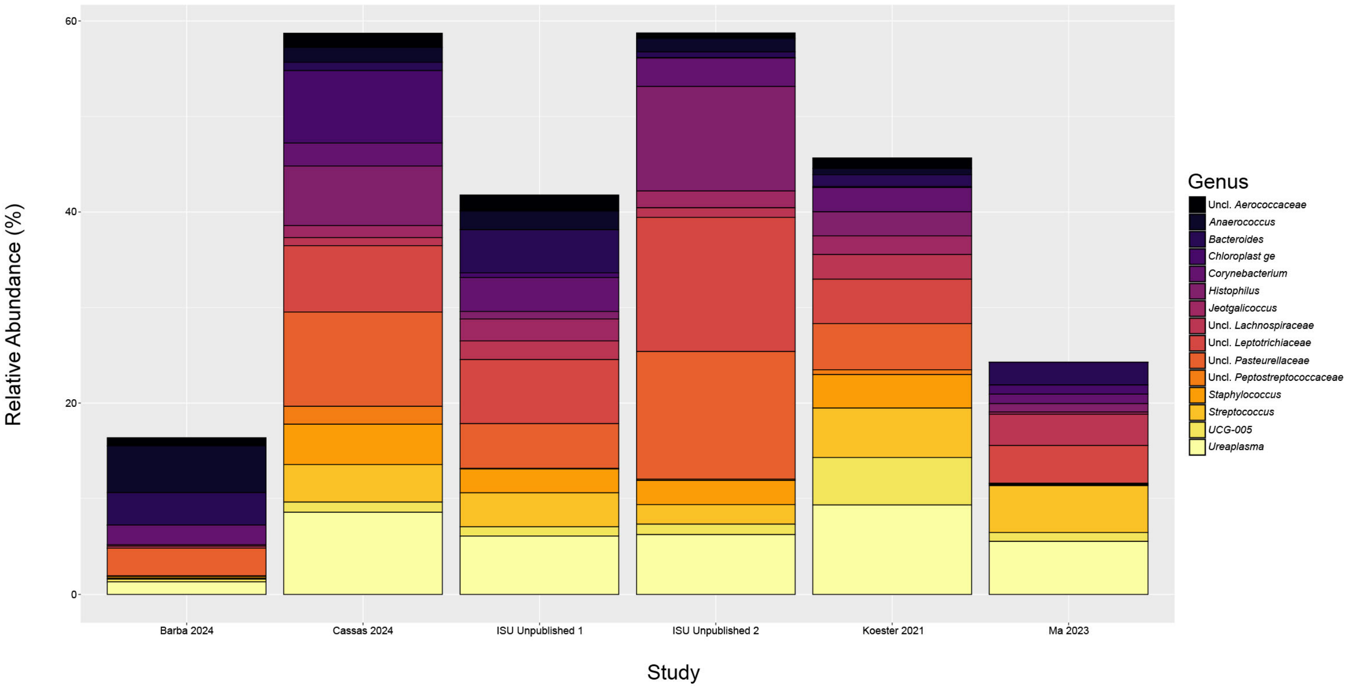

Additional file 5: S2 Figure: Relative abundance of the 15 most abundant genera in the sheep vaginal microbiota.
