# Supplementary material for: Combined analysis of 16S rRNA gene sequencing data reveals core vaginal bacteria across livestock species
Source: Front Microbiol. 2025 Feb 10;16:1524000. doi: 10.3389/fmicb.2025.1524000 (PMC11849051; doi:10.3389/fmicb.2025.1524000)
Supplement: Supplementary file 5 [file Table_2.DOCX]

**Supplementary Table 2: Relative abundance (in percent) of the 10 most abundant phyla across all samples**

| **Phylum** | **All samples** | **Cattle samples** | **Sheep samples** | **Pig samples** |
| --- | --- | --- | --- | --- |
| *Bacillota* | 49.79 | 58.75 | 42.59 | 50.42 |
| *Pseudomonadota* | 19.11 | 9.33 | 23.38 | 21.22 |
| *Bacteroidota* | 12.34 | 18.43 | 7.67 | 12.61 |
| *Fusobacteriota* | 6.74 | 1.02 | 8.04 | 8.91 |
| *Actinobacteriota* | 6.05 | 6.39 | 8.46 | 3.97 |
| *Cyanobacteria* | 2.00 | 0.57 | 5.42 | 0.11 |
| *Campilobacterota* | 0.89 | 0.54 | 0.88 | 1.09 |
| *Euryarchaeota* | 0.78 | 1.48 | 0.71 | 0.45 |
| *Spirochaetota* | 0.64 | 0.38 | 0.80 | 0.67 |
| *Verrucomicrobiota* | 0.48 | 1.25 | 0.39 | 0.13 |
