# Supplementary material for: Combined analysis of 16S rRNA gene sequencing data reveals core vaginal bacteria across livestock species
Source: Front Microbiol. 2025 Feb 10;16:1524000. doi: 10.3389/fmicb.2025.1524000 (PMC11849051; doi:10.3389/fmicb.2025.1524000)
Supplement: Supplementary file 6 [file Table_3.DOCX]

**Supplementary Table 3: Relative abundance (in percent) of the 50 most abundant genera across all samples**

| **Genus** | **All samples** | **Cow samples** | **Sheep samples** | **Pig samples** |
| --- | --- | --- | --- | --- |
| Unclassified Pasteurellaceae | 8.30 | 0.96 | 8.85 | 11.97 |
| Ureaplasma | 5.05 | 9.27 | 8.49 | 0.00 |
| Streptococcus | 4.36 | 2.19 | 4.01 | 5.85 |
| Fusobacterium | 4.17 | 0.34 | 0.86 | 8.90 |
| Clostridium_sensu_stricto_1 | 4.07 | 1.87 | 0.60 | 8.02 |
| Corynebacterium | 2.53 | 3.05 | 2.46 | 2.30 |
| Histophilus | 2.49 | 2.74 | 5.48 | 0.00 |
| UCG-005 | 2.44 | 6.48 | 1.72 | 0.74 |
| Unclassified Leptotrichiaceae | 2.42 | 0.48 | 6.89 | 0.00 |
| Bacteroides | 2.03 | 2.49 | 1.16 | 2.47 |
| Unclassified Lachnospiraceae | 1.93 | 2.50 | 1.26 | 2.14 |
| Unclassified Enterobacteriaceae | 1.92 | 0.48 | 0.19 | 4.09 |
| Romboutsia | 1.91 | 3.96 | 0.58 | 1.80 |
| Staphylococcus | 1.89 | 0.31 | 3.87 | 1.22 |
| Parvimonas | 1.87 | 0.04 | 0.32 | 4.11 |
| Prevotella | 1.85 | 0.58 | 0.62 | 3.52 |
| Chloroplast_ge | 1.81 | 0.21 | 5.22 | 0.04 |
| Anaerococcus | 1.58 | 0.12 | 1.35 | 2.58 |
| Turicibacter | 1.38 | 0.79 | 0.74 | 2.21 |
| UCG-010_ge | 1.36 | 4.84 | 0.45 | 0.14 |
| Porphyromonas | 1.21 | 0.61 | 0.77 | 1.89 |
| Rikenellaceae_RC9_gut_group | 1.17 | 3.36 | 0.60 | 0.40 |
| Christensenellaceae_R-7_group | 0.99 | 2.19 | 0.87 | 0.41 |
| Unclassified Aerococcaceae | 0.82 | 1.03 | 1.32 | 0.31 |
| Terrisporobacter | 0.82 | 0.09 | 0.02 | 1.84 |
| Campylobacter | 0.80 | 0.51 | 0.73 | 1.03 |
| Lactobacillus | 0.75 | 0.47 | 0.42 | 1.17 |
| Veillonella | 0.74 | 0.01 | 0.02 | 1.71 |
| Methanobrevibacter | 0.72 | 1.36 | 0.63 | 0.44 |
| Oscillospirales_ge | 0.71 | 2.00 | 0.38 | 0.25 |
| Unclassified Peptostreptococcaceae | 0.71 | 0.93 | 1.43 | 0.03 |
| Peptoniphilus | 0.71 | 0.34 | 0.91 | 0.76 |
| Treponema | 0.61 | 0.34 | 0.75 | 0.66 |
| Unclassified Bacteroidales | 0.58 | 1.02 | 0.28 | 0.58 |
| Jeotgalicoccus | 0.55 | 0.06 | 1.44 | 0.12 |
| Acinetobacter | 0.55 | 0.41 | 0.52 | 0.64 |
| Prevotellaceae_UCG-003 | 0.53 | 1.55 | 0.30 | 0.14 |
| Alistipes | 0.52 | 1.73 | 0.32 | 0.01 |
| Bifidobacterium | 0.51 | 0.78 | 0.80 | 0.12 |
| Paeniclostridium | 0.50 | 1.96 | 0.11 | 0.00 |
| Aerococcus | 0.50 | 0.25 | 0.13 | 0.92 |
| Unclassified Staphylococcaceae | 0.44 | 0.09 | 1.04 | 0.16 |
| Peptostreptococcus | 0.43 | 0.02 | 0.07 | 0.95 |
| Clostridia | 0.43 | 0.79 | 0.14 | 0.45 |
| Trueperella | 0.43 | 0.29 | 0.82 | 0.19 |
| Unclassified Ruminococcaceae | 0.40 | 0.76 | 0.46 | 0.16 |
| Alloprevotella | 0.40 | 0.48 | 0.28 | 0.46 |
| Yersinia | 0.39 | 0.01 | 0.00 | 0.91 |
| Finegoldia | 0.39 | 0.07 | 0.56 | 0.44 |
| Helcococcus | 0.39 | 0.61 | 0.17 | 0.43 |
